# Predictive Biomarkers to Chemoradiation in Locally Advanced Rectal Cancer

**DOI:** 10.1155/2015/921435

**Published:** 2015-10-04

**Authors:** Raquel Conde-Muíño, Marta Cuadros, Natalia Zambudio, Inmaculada Segura-Jiménez, Carlos Cano, Pablo Palma

**Affiliations:** ^1^Division of Colon & Rectal Surgery, University Hospital Virgen de las Nieves, Spain; ^2^Department of Biochemistry, University of Granada, Spain; ^3^Department of Computer Science and Artificial Intelligence, University of Granada, Spain

## Abstract

There has been a high local recurrence rate in rectal cancer. Besides improvements in surgical techniques, both neoadjuvant short-course radiotherapy and long-course chemoradiation improve oncological results. Approximately 40–60% of rectal cancer patients treated with neoadjuvant chemoradiation achieve some degree of pathologic response. However, there is no effective method of predicting which patients will respond to neoadjuvant treatment. Recent studies have evaluated the potential of genetic biomarkers to predict outcome in locally advanced rectal adenocarcinoma treated with neoadjuvant chemoradiation. The articles produced by the PubMed search were reviewed for those specifically addressing a genetic profile's ability to predict response to neoadjuvant treatment in rectal cancer. Although tissue gene microarray profiling has led to promising data in cancer, to date, none of the identified signatures or molecular markers in locally advanced rectal cancer has been successfully validated as a diagnostic or prognostic tool applicable to routine clinical practice.

## 1. Introduction

Colorectal cancer is the third most frequent cancer and the second most frequent cause of cancer related death, both in Europe [1]. The proportion of rectal cancer cases is variable depending on the cancer registry and classification of rectosigmoid tumours, ranging from 27% to 58% [2]. The ideal treatment recommendations for rectal cancer are under permanent appraisal; nevertheless, studies have demonstrated that, for locally advanced rectal cancer (LARC) (stage T3, stage T4, or node-positive disease), preoperative (neoadjuvant) chemoradiation (CRT) significantly improves local control and reduces toxicity profiles compared with postoperative CRT but with similar survival rates [3, 4]. Furthermore, the ability to achieve pathologic downstaging, or a complete pathologic response (pCR), after neoadjuvant CRT is correlated with improved survival, decreased local recurrence, and a higher rate of sphincter-preserving surgeries [5]. Approximately 40–60% of LARC patients treated with neoadjuvant CRT achieve some degree of pathologic response. However, there is no effective method of predicting which patients will respond to neoadjuvant CRT [6]. Prospective identification of patients who have a higher likelihood of responding to preoperative CRT could be important in deceasing treatment morbidity and improving survival and local control in LARC. In addition, patients who are unlikely to respond could be offered alternative approaches to therapy. Recent studies have evaluated the potential of genetic biomarkers to predict outcome in LARC treated with neoadjuvant CRT [7, 8]. The goal of this review is to examine the current literature for the most commonly researched biomarkers for predicting outcome to neoadjuvant CRT in LARC patients.

## 2. Material and Methods

An exhaustive search of PubMed was performed on March, 2014, with combinations of the following terms: “rectal cancer,” “response,” “prediction,” “microarray,” “gene expression,” “mi-RNA,” and “ln- RNA.” The articles produced by the PubMed search were reviewed for those specifically addressing a genetic profile's ability to predict response to neoadjuvant CRT in LARC (genes, microRNA, or long noncoding RNA). Articles analysing response prediction to CRT in colorectal cancer cell lines were excluded. Sixteen studies evaluating genetic profiles predicting outcome of neoadjuvant CRT in rectal cancer were found. Ten of them identified an over- or downregulated gene signature, 5 found microRNA (miRNA) signature. Only one screened long noncoding RNA (lncRNA) was associated with radiosensitivity but was made in colorectal cancer cell lines and was written in Chinese and therefore was excluded.

## 3. Results

### 3.1. Prediction of Response Based on DNA Microarrays in Tumor Tissue (prior to Neoadjuvant Treatment) (Table 1)

The first study on the application of a genetic signature to predict response to neoadjuvant treatment in rectal cancer appeared in 2005 [9]. It included 30 patients from a data base pertaining to the German Group for the Study of Rectal Cancer [22] who received preoperative chemoradiation therapy (50.4 Gy of radiation, applied in 28 fractions and continuous infusion of 5FU). They underwent surgery 6 weeks following completion of the neoadjuvant therapy. Response to treatment was measured by the following: tumor shrinkage (when compared with a preoperative ultrasound scan, uT) and the stages of tumor remission under Dworak's regression grades (3-4 considered to be responders) [23]. Based on* downsizing* or tumor shrinkage they identified 54 genes expressed differently between responders versus nonresponders in tumor samples extracted prior to neoadjuvant therapy. By using these genes they attained 83% precision in the prediction, both for responders and nonresponders, thus proving that the study of genetic expression through* microarrays* was useful in predicting a reduction in tumor size (measured by the decrease of ypT in relation to uT) in response to preoperative CRT therapy. These 54 genes are involved in many biological functions, including repairing damage to cellular DNA (SMC1), organizing microtubules (CLMN and CDC42BPA), and cellular signaling (FLNB).

The following year a Japanese group with a similar objective, published a* microarray* analysis of DNA [10] that analyzed a total of 52 patients. Neoadjuvant therapy consisted of preoperative radiotherapy (50.4 Gy) without any chemotherapy, followed by a four-week rest period and then surgery. The evaluation of response was determined based on an anatomical pathological analysis of the surgical sample, employing a Japanese semiquantitative scale that identified stages 2-3 as responders and stages 0-1 as nonresponders. A group of 33 differentially expressed genes was established among responders and nonresponders: 20 were overexpressed genes related to apoptosis such as lumican (LUM), thrombospondin 2 (THBS2), and galectin-1 (LGALS1), while 13 were repressed in the responder-group, such as cyclophilin 40 (CYP40) and glutathione peroxidase 2 (GPX2). A protein structure prediction was then done on 33 genes from 17 patients included in the validation group, which found 82.4% exactness for determining class, 50% sensibility, 100% specificity, a positive predictive value of 100%, and a negative predictive value of 76.6%.

Kim and colleagues conducted a study in 2007 using samples from 46 patients (31 for the initial trial group and 15 for the validation) [11]. Neoadjuvant treatment included radiotherapy (50.4 Gy in 28 fractions) and chemotherapy (5FU + leucovorin, capecitabine or capecitabine + irinotecan). Patients underwent surgery 6 weeks after completion of treatment; tumor response was classified according to Dworak's tumor regression grade system. They identified a group of 95 genes and applied the* leave-one-out-cross-validation *(LOOCV) method to predict response and found that this group of genes enabled tumor response to be predicted with 84% precision, 64% sensibility, 95% specificity, an 88% positive predictive value and an 87% negative predictive value. The validation group reached 87% precision, 100% sensibility, and 82% specificity. Two of the 95 genes stood out: thymidylate synthase (TYMS, involved in DNA synthesis), which was highly expressed in responding tumors, and RAD23B (involved in nucleotide excision repair), which was elevated in nonresponders and has previously been associated with patients resistant to treatment with 5FU. These two genes could be used to evaluate response to treatment with 5FU.

Rimkus et al. [12] also studied the tumor biopsies of patients in stage T3. The therapeutic approach used involved radiation (45 Gy) and continuous infusion of 5FU. Surgery was done following a 4–6-week rest period. The anatomical pathological response was classified according to Becker's regression grade (responders in stage 1 and nonresponders in stages 2-3). They found 42 statistically significant genes that were expressed differently among responders and nonresponders. Five of them (FREM1, M-RIP, SDHC, TDE1, and USP42) had a reduced expression in the group of responders, while the rest of the genes were overexpressed and involved in apoptosis (CASP1), transport (SLC35E1), cellular signaling (STAT2 and ETS2), and cellular cycle (CCNK). Sensibility was 71%, specificity was 86%, positive predictive value was 71%, and negative predictive value was 86%.

More recently, a group formed by Nishioka and colleagues [13] included 20 patients (17 in a trial group and 3 in the validation unit) who received radiotherapy (40 Gy in fractions of 2 Gy), associated with S1, an oral chemotherapeutic agent whose action is similar to capecitabine, not currently authorized for use in Europe. They used a response scale from the Japanese Society for Cancer of the Colon and Rectumthat classified patients in groups 0-1 as nonresponders and those in groups 2-3 as responders. A* microarray* of 132 genes related to a response to 5FU was used in addition to other chemotherapeutics. Researchers identified 17 genes expressed differently among the two patient subgroups (responders versus nonresponders). Of them, five were metalloproteinases (MMP1, MMP7, MMP9, MMP14, and MMP16). In addition, they conducted an immunological and histological chemical study to evaluate expression of the MMP7 protein, whose gene was the one most overexpressed in normal tissue. Among responders, four patients overexpressed MMP7 (4/10, 40%), while none in the group of nonresponders expressed that protein. These 17 genes were used to classify samples from the validation group and were correct in three cases. It is worth emphasizing that in the nonresponder case none of the genes were overexpressed.

Our group [7] identified a 4-gene profile (C-MYC, GNG4, POLA, and RRM1) associated with response to preoperative chemoradiotherapy in rectal cancer patients. The microarrays study included a total of 35 patients with LARC with additional 8 patients in the validation group. Tumor samples were prospectively obtained before treatment (total dose of 50.4 Gy of radiation in 28 fractions of 1.8 Gy associated with capecitabine alone or capecitabine combined with oxaliplatin). Tumor response was assessed in surgical specimens by pathological examination based on Mandard's tumor regression grading (TRG) system [24]: TRG 1 and TRG 2 scores were considered responders, whereas TRG 3, TRG 4, and TRG 5 scores were classified as nonresponders. To validate microarray experimental data, expression levels of 20 genes in rectal tumor patients were obtained by real-time quantitative reverse transcription PCR. 257 genes were overexpressed in responders, but only 4 were confirmed with PCR. High expression levels of the 4 genes (Gng4, c-Myc, Pola1, and Rrm1) were a significant prognostic factor for response to treatment in LARC patients. Using this gene set, a new model for predicting the response to CRT in rectal cancer was established with a sensitivity of 60% and 100% specificity. For each of the genes Gng4, c-Myc, Pola1, and Rrm1, receiver operating characteristic (ROC) curves were computed. The one with the higher AUC was c-Myc, with a sensitivity of 70% and a specificity of 100% at a cut-off point set at 64.45. Functional analysis showed that the encoding proteins were associated with several canonical pathways (pyrimidine and purine metabolism, colorectal cancer metastasis signalling). The most significant network consisting of 49 genes contained 24 focus genes directly or indirectly connected to a c-Myc network.

Granttet published a study in 2014 using high-throughput nucleotide* microarrays* to develop a genetic profile associated with CRT-resistant rectal cancer. Thirty-three patients were incorporated in the study [15]. Patients who met clinical criteria for neoadjuvant CRT underwent biopsy of the tumour. The treatment regimen included 50.4 Gy radiation in 30 fractions with 5-fluorouracil. Patients underwent curative surgery approximately 8–10 weeks after completion of CRT. Posttreatment responses were assessed according to the American Joint Committee on Cancer (AJCC) criteria considering patients with AJCC 0–2 as responders and those with AJCC 3 as nonresponders. They identified a unique gene expression profile composed of 812 genes associated with rectal cancer that had a poor response to CRT. This profile enabled the classification of nonresponders with 100% accuracy in a small validation group (sensitivity and specificity of 100% for predicting nonresponders). Using the 183-gene profile, specificity remained 100%, while sensitivity decreased to 33.3%. The top 10 upregulated genes included APOA2, AHSG, DBH, APOA1, APOB, APOC3, LMX1A, SOAT2, SLC7A9, and TF. The top 10 downregulated genes included LOC729399, SERINC5, SCNN1B, ZC3H6, SLC4A4, DTWD2, MS4A12, BEX5, MMRN1, and CLCA4. Functional analysis of differentially expressed genes with IPA software (Ingenuity Pathways Analysis) revealed “DNA repair by homologous recombination” as a statistically significant canonical pathway in this study with RAD50 as the most significant differentially expressed gene in this pathway. RAD50 is a member of the MRE11-RAD50-NBS1 (MRN) complex that detects double-stranded DNA breaks and regulates DNA damage repair primarily through homologous recombination. A number of apolipoprotein genes were upregulated in nonresponders (APOA2, APOA1, APOB, and APOC3). AHSG is a serum glycoprotein involved in endocytosis, brain development, and the formation of bone tissue previously associated with resistance to neoadjuvant chemotherapy in patients with advanced breast cancer. LMX1A is known to be involved in insulin gene transcription and the embryogenesis of dopamine-producing neurons. In cancer, LMX1A has been shown to be a poor prognostic indicator in ovarian and pancreatic tumors but LMX1A was also recently shown to inhibit cell proliferation, migration, invasion, and colony formation in vitro.

Recently, Watanabe conducted a new study to establish a prediction model for response to chemoradiotherapy in rectal cancer based on gene expression by RT-PCR analysis as it allows accurate and reproducible quantification of genes [16]. Biopsy specimens were collected before preoperative treatment (50.4 Gy in 28 fractions during 6 weeks concomitantly with tegafur-uracil and leucovorin). Standardized curative resection was performed 6 weeks after the completion of chemoradiotherapy. Response to chemoradiotherapy was determined by histopathological examination of surgically resected specimens based on a semiquantitative classification system defined by the Japanese Society for Cancer of the Colon and Rectum. Tumors were classified as “nonresponders” when assigned to grade 0 or grade 1 and “responders” when assigned to regression grade 2 or grade 3. First, gene expression profiles were determined by DNA microarray analysis on 46 training samples. They identified 24 probes that were differentially expressed between responders and nonresponders. Twenty genes showed higher and four genes showed lower expression in nonresponders compared with responders. Microarray expression levels were validated by quantitative RT-PCR of 18 genes (that were represented among the 24 probes) in the 46 training samples, showing significant differences in the expression levels of 16 of the 18 genes (20 probes) between responders and nonresponders. Based on the 16 genes and their combination, the predictive accuracies of the 2500 different sets of predictor genes were calculated. The highest accuracy rate (89.1%) was obtained with a 4-gene set including LRRIQ3, FRMD3, SAMD5, and TMC7. This 4-gene signature was validated in an independent cohort of 16 patients. Predictive accuracy rate was 81.3% and sensitivity, specificity, positive predictive value, and negative predictive value were 100%, 62.5%, 72.7%, and 100%, respectively.

### 3.2. Prediction of Response Based on* Microarrays *of Gene Expression in Peripheral Blood

Peripheral blood mononuclear cells have emerged recently as pathology markers of cancer and other diseases, making their use as therapy predictors possible. Furthermore, the importance of the immune response in radiosensitivity of solid organs led Palma et al. [8] to hypothesize that microarray gene expression profiling of peripheral blood mononuclear cells could identify patients with response to CRT in LARC. Thirty-five 35 patients with locally advanced rectal cancer were recruited initially to perform the study. Peripheral blood samples were obtained before neoadjuvant treatment. RNA was extracted and purified to obtain cDNA and cRNA for hybridization of microarrays included in Human WG CodeLink bioarrays. Quantitative real-time PCR was used to validate microarray experiment data. Results were correlated with pathological response, according to Mandard's criteria and final UICC Stage (patients with tumor regression grades 1-2 and downstaging being defined as responders and patients with grades 3–5 and no downstaging as nonresponders). The authors performed a multiple *t*-test using Significance Analysis of Microarrays to find those genes differing significantly in expression between responders (*n* = 11) and nonresponders (*n* = 16) to CRT. The differently expressed genes were BC 035656.1, CIR, PRDM2, CAPG, FALZ, HLA-DPB2, NUPL2, and ZFP36. The measurement of FALZ (*P* = 0.029) gene expression level determined by qRT-PCR showed statistically significant differences between the two groups. They postulated the idea that gene expression profiling reveals novel genes in peripheral blood samples of mononuclear cells that could predict responders and nonresponders to CRT in patients with LARC. The authors hypothesized the importance of mononuclear cells' mediated response in the neoadjuvant treatment of rectal cancer.

### 3.3. Prediction of Response Using* Microarrays* of MicroRNA (Table 2)

MicroRNAs (miRNAs), discovered in 1993, represent a relatively new field in the rapidly developing world of genetics and the regulation of genetic expression. A miRNA is a small sequence of single-stranded RNA (normally between 18 and 25 nucleotides) that do not code proteins but do act as posttranscriptional regulators of genetic expression. They act by binding to complementary strands of messenger RNA, usually inhibiting expression and silencing the gene. Their function can be very similar to the function of oncogenes as well as tumor-suppressing genes [25]. The aberrant expression of miRNA is involved in numerous pathologies and some alterations in its regulation have been associated with colorectal cancer. Furthermore, it has been determined that CRT in LARC can induce alterations in the expression of miRNA in normal tissue samples and these have been associated with positive response to treatment [26].

Changes in miRNA expression can be induced as a consequence of various external stimuli such as hypoxia and gemcitabine. Svoboda and colleagues studied changes of selected microRNAs in rectal cancer biopsies from patients treated with chemoradiotherapy (50.4 Gy in 1.8 fractions concomitantly with capecitabine) and correlation with response [17]. Microexcision biopsies were taken from the same rectal cancers before therapy and subsequently two weeks after starting preoperative chemoradiotherapy treatment. Radical surgery was performed within the 6th week after completion of neoadjuvant treatment. Tumor response to therapy was assessed microscopically by the Dworak tumor regression grade system. Following a pilot study of normal mucosa biopsies researchers found that microRNAs mi-R125b and mi-R137 showed significant induction and exhibited the same expression trends in most samples two weeks after starting therapy, so they were chosen for further analysis in the total sample set. Real-time PCR was performed and relative expressions of microRNA were determined. Patients with early tumors have lower induction than patients with higher stage cancers. MiR125b is downregulated in several cancers and thought to act as a tumor suppressor. In this study, tumors with the highest upregulation of mi-R125b level two weeks after starting therapy showed no downstaging and less regression (poor response). Mi-R137 was significantly upregulated only in the most advanced T-stage. Researchers concluded that higher induced levels of mi-R125b and mi-R137 were associated with a worse response to the therapy.

In 2012 Della Vittoria Scarpati and colleagues published an article based on this technique which established a specific profile associated with response to treatment in the biopsies of patients with locally advanced rectal neoplasms who underwent neoadjuvant therapy [18]. The team took biopsies from 35 patients affected by rectal cancer T3-4/N+ prior to the initiation of radiotherapy (45 Gy) combined with capecitabine and oxaliplatin. Following a 6-to-8-week rest period a conventional surgical resection was performed. The anatomical pathological response was classified according to Mandard's tumor regression scale: responding patients (TRG 1) and nonresponders (TRG 2, TRG 3, TRG 4, and TRG 5). Results were then validated through quantitative RT-PCR. Researchers studied 373 miRNAs, 53 of which were overexpressed in the group of responders compared with 4 in the group of nonresponders. Of those, 14 were selected for validation by RT-PCR and 13 of them were confirmed. Two of the miRNAs involved in DNA repair mechanisms stood out (miR-622 and miR-630), possibly inhibiting the process and converging in the P53 pathway. These two miRNAs are not expressed in samples proceeding from responding patients and show 100% sensibility and sensitivity.

The authors of another recently published study [19] extracted 12 RNA samples from pretherapeutic biopsies embedded in paraffin and then compared their RNA expression profile with response to neoadjuvant chemoradiation. They identified three RNAs associated with complete response (miR-16, miR-153, and miR-590-5p), employing Mansard's tumor regression grade for quantification and two (miR-519c-3p and miR-561) that predicted good versus poor response, with exactness close to 100%. miRNA expression was analysed in formalin-fixed paraffin-embeddedsamples and in fresh-frozen samples using real-time PCR. The expression levels of miR-10b, miR-143, and miR-145 were downregulated in both FFPE and fresh-frozen tissues, while those for miR-21 were upregulated in tumors.

In another retrospective study large-scale miRNA expression analysis was performed on 20 samples of preoperative biopsies of rectal cancer tissues [20]. All patients underwent neoadjuvant treatment based on radiotherapy (45 Gy to the pelvis plus 5.4 Gy boost to tumor) and chemotherapy with capecitabine or 5FU followed 6 weeks later by standard radical surgery. Response was evaluated using a grading system adapted from Mandard and establishing an average and a maximal percentual representation of residual cancer cells in the cell population detected in 10 examined slices of formalin-fixed and paraffin-embedded primary tumors. Responders were classified as patients with tumors in TRG 1-2 and nonresponders were those with or without partial regression (TRG 3–5). Researchers identified eight miRNAs with different expression levels between the two groups. Three of them (miR-215, miR190b, and miR-29b-2) were overexpressed, while the other five (let7e, miR-196b, miR-450a, miR-450b-5p, and miR-99a) showed lower expression levels in nonresponders. Using these miRNAs, 90% of responders and 90% of nonresponders were correctly classified. Five of them (miR-215, miR-99a^*∗*^, miR-196b, miR-450b-5p, and let-7e) were previously correlated with radioresistance or chemoresistance to thymidylate synthase inhibitors. There is evidence in previous studies that MiR-215 induces inhibition of cell proliferation and subsequent chemoresistance. The let-7 family of miRNAs (let-7a through let-7h) regulates expression of key oncogenes, such as RAS and MYC, and is specifically downregulated in many cancer types. Important proteins involved in DNA repair are among putative targets of miR-99a^*∗*^, so upregulation of miR-99a^*∗*^ in tumors could be associated with lower DNA repair capacity through downregulation of these genes, which may lead to radiotherapy sensitization. Researchers concluded that miRNAs are part of the response mechanism involved in rectal cancer to chemoradiotherapy and that miRNAs could represent promising predictive biomarkers for patients undergoing such treatment.

Hotchi's group from Japan obtained rectal cancer samples during colonoscopy from 43 patients, prior to preoperative chemoradiotherapy (22 for training and 21 for testing the outcome prediction model) [21]. Samples were used for RNA extraction when paralleled biopsies contained at least 70% tumor cells. Neoadjuvant treatment consisted of 4,000 cGy of pelvic irradiation, five times a week, with a daily fraction of 200 cGy utilizing a four-field technique concomitantly with S1 on radiation days (a novel oral fluoropyrimidine inhibitory for dihydropyrimidine dehydrogenase with a potent radiosensitizing property). Surgery was performed 6–8 weeks following completion of preoperative CRT. Response to CRT was evaluated by three parameters:

1. Histopathological examination of surgically resected specimens (based on a semiquantitative classification system). Tumors were classified as responders when assigned to regression grade 2 or grade 3 and nonresponders when assigned to grade 0 or grade 1.
2. Response Evaluation Criteria in Solid Tumors (RECIST): tumors were classified as responders when assigned to complete response (CR) or partial response (PR) and nonresponders when stable disease (SD) or progressive disease (PD) was reported.
3. Downstaging (yes/no): using real-time RT-PCR in a training set, a candidate miRNA detected by miRNA microarray analysis was evaluated.

With regard to the histopathological examination of surgically resected specimens, two genes are differentially expressed at significant levels in responders and nonresponders (miR-223 and miR-142-3p), with responders showing higher expression in comparison to nonresponders. Nine genes were differentially expressed at significant levels with regard to RECIST: one (miR-223) showed a higher expression, while eight showed a lower expression (miR-20b, miR-92a, let-7a^*∗*^, miR-20a, miR-17^*∗*^, miR-106a, miR-17, and miR-20a^*∗*^) in responders compared to nonresponders. Three genes (miR-223, miR-630, and miR-126^*∗*^) showed a higher expression in responders compared to nonresponders with regard to downstaging. A candidate gene, miR-223, showed a higher expression among responders than nonresponders in the three parameters evaluated using real-time RT-PCR. The miR-223 level was significantly higher in responders compared to nonresponders. ROC curve analyses showed that miR-223 might differentiate between responders and nonresponders with an area under the curve (AUC) of 0.768 (95% confidence internal (CI), 0.661–0.865). At the cut-off value of 0.4 for miR-223, the sensitivity and the specificity in the 21 testing samples were 100 and 78.0%, respectively.

### 3.4. Response Prediction Using SAGE (Serial Analysis of Gene Expression)

In 2011 Casado and colleagues performed a serial analysis of genetic expression to identify a genetic profile that could predict response to chemoradiation therapy in locally advanced rectal cancer [14]. An initial selection of genes was made using SAGE analysis. They recruited 25 patients and applied a neoadjuvant therapy regimen composed of oxaliplatin and raltitrexed (130 mg/m^2^ and 3 mg/m^2^, days 1, 21, and 42) in three cycles, combined with radiotherapy (50.4 Gy in 28 fractions). Response was determined in the surgical sample following the scale used by Dworak and colleagues [23]. In contrast to studies presented to date, the goal here was to find genes predictive of a poor response. They identified 24 genes associated with a lack of response. Based on these results and available literature, the team selected 53 genes for a subsequent retrospective study in 94 patients with locally advanced rectal cancer that had received neoadjuvant treatment (under four different radiochemotherapy regimens). They used stored samples of those tumors embedded in paraffin and performed a qRT-PCR following the* TaqMan Low Density Array *(TLDA) protocol. This enabled them to identify a genetic profile composed of 13 genes that permitted the prediction of nonresponse with an exactness of 85%, sensibility of 87%, and specificity of 82%. This study's weakest point is the diversity of neoadjuvant treatments employed. Technology based on tumor samples embedded in paraffin to determine genetic profiles is more suitable to clinical practice than the use of* microarray* studies of gene expression.

Currently, a multicentric study backed by the* Grupo Español Multidisciplinar en Cancer Digestivo* (Multidisciplinary Spanish Group on Digestive Cancer, GEMCAD) is underway to confirm these results.

## 4. Discussion

There has been a high local recurrence rate in LARC. Besides improvements in surgical techniques, both neoadjuvant short-course radiotherapy and long-course chemoradiation improve oncological results [27]. After CRT, the ability to achieve tumor reduction or even a pCR is observed in up to 60% of the patients treated. This treatment also correlates with a decreasing local recurrence. Conversely patients with a poor response have a worse oncological outcome.

Modern oncological treatment decisions increasingly depend on so-called clinical and laboratory predictive and prognostic markers. While prognostic markers explain variability irrespective of treatment, our study intends to use predictive markers to explain outcome variability in response to treatment. Gene expression profile using the microarray technology has led to a series of promising results through tissue gene expression profiling of different malignancies, including cancer. Interestingly, gene signatures have been used successfully as prognostic predictor for patients with colorectal carcinomas [9, 28].

A successful biomarker should be able to predict a certain group of rectal cancer patients that would be likely to experience response or even a pCR. For this group of patients, the biomarker would be a useful prognostic factor that could indicate a more favorable outcome, and their management would not change from the standard treatment regimen. Those patients with biomarkers predicting a poor response to standard treatment could be offered adjusted therapy courses in terms of the agents used or sequence of treatments (e.g., induction chemotherapy, the addition of a targeted agent such as an EGFR antibody, or surgery without any delay, followed by adjuvant CRT).

The literature was reviewed for studies of biomarkers predicting response to neoadjuvant CRT for rectal cancer. Fifteen studies evaluating genetic profiles predicting outcome of neoadjuvant CRT in rectal cancer were analyzed. Ten of them identified an over- or downregulated gene signature; five studies found microRNA (miRNA) signature.

Although tissue gene microarray profiling has led to promising data in cancer, to date, none of the identified signatures or molecular markers in LARC has been successfully validated as a diagnostic or prognostic tool applicable to routine clinical practice. Moreover, there has been little agreement between signatures published, with scarce overlap in the reported genes [9–13]. Only two genes, MMP4 and FLNA, have been reported in more than one paper [10, 11, 13] and only one of the 257 genes reported by our research group, RRM1 (an important marker for chemotherapy resistance in colon tumors [29]), was also identified by Nishioka et al. [13].

Significant bias was found by analyzing the literature. The scant number of patients in the studies is one of them. The evaluated studies examined between 12 and 94 patients. Even if a significant correlation was determined between a biomarker and a measurement of outcome, the literature has failed to demonstrate reproducibility. Before the clinical use can be established, prospective studies, including a large number of patients should be performed in order to achieve reproducible results.

Furthermore, significant variability in the CRT course can hinder the interpretation of results. Neoadjuvant CRT for LARC typically consists of 5FU and 45–50.4 Gy of pelvic irradiation. By using alternative chemotherapeutic agents in the studies, the results are more difficult to interpret. For example, the addition of oxaliplatin or irinotecan to 5FU for a subset of patients could confound the outcome measurements by altering the baseline response. Variability in the response scoring system is also a debatable bias between the studies.

Despite this variability, our review underlines two main hypothesis: first, the elevated expression of c-Myc mRNA as an important marker of response to CRT in LARC as an essential component of the neoplastic phenotype in rectal tumors.

Second, miRNAs, highly conserved noncoding RNAs ranging between 21 and 24 nucleotides in size, play a major role in the posttranscriptional regulation of mRNA. The inhibition of translation after forming a complex similar to the RNA-interference-induced silencing complex (RISC), by downregulating the expression of their protein-coding gene targets, is the general mechanism of microRNA action in animal and human cells [30, 31]. Some miRNAs function as oncogenes, while others could function as tumor suppressors. miRNAs are considered to be master regulators of several important biological processes, such as cell growth, apoptosis, and cancer development [32–35]. miRNA expression profiles have been shown to be promising biomarkers for the classification or the outcome prediction of some human cancers [35, 36]. Moreover, miRNAs are involved in different stages of colorectal cancer pathogenesis by regulating the expression of oncogenes and tumor suppressor genes [37]. They are also known to be involved in the regulation of radioresistance [38, 39]. Due to their small size, miRNAs are more stable and resistant to environmental, physical, and chemical stresses compared to mRNAs. Therefore, their analysis as formalin-fixed paraffin-embedded tissue samples may provide more accurate replication of what would be observed in fresh tissues [19]. The analysis of formalin-fixed paraffin-embedded samples could be easily transferred to clinical practice.

In conclusion, the current literature does not lend enough support to any of the biomarkers to permit the clinical application in order to predict outcome to neoadjuvant CRT in rectal cancer.

In future clinical trials, assessing neoadjuvant CRT for rectal cancer, these biomarkers should be prospectively evaluated to determine their role as predictors of outcome. It is clear that there is a biological basis as to why some tumors respond to CRT and that biology could be related to the tumor, the patient, or both. In this context, the genes identified in Mononuclear Peripheral Blood Cells could offer new insights into the immune system's dysregulation in LARC [8] and should be further investigated. Furthermore, the answer may not lie strictly in the genome of the tumor but could represent epigenetic factors, and these would also need to be explored. It is unlikely that any single factor will determine response characteristics; therefore a multifaceted approach will almost certainly be needed.

## Figures and Tables

**Table 1:** Studies showing DNA microarray gene expression profile predictive of response to CRT in LARC.

Study	Specimen	*N* patients	Validation group	*Radiotherapy dose* Chemotherapy	Response assessment	Identified genes: more relevant genes	Outcome
Ghadimi et al. 2005 [9]	Tumor tissue biopsy	30	No	*50.4 Gy/28 fractions* 5FU	*Downsizing*	54 genes: SMC1, CLMN, CDC42BPA, and FLNB	Group prediction 83%

Watanabe et al. 2006 [10]	Tumor tissue biopsy	52	17	*50.4 Gy* —	Japanese Classification of Colorectal Carcinoma	33 genes (i) Overexpressed: LUM, THBS2, and LGALS1 (ii) Downregulated: CYP40 and GPX2	Class prediction 82.4%, sensitivity 50%, specificity 100%, PPV 100%, NPV 76.6%

Kim et al. 2007 [11]	Tumor tissue biopsy	31	15	*50.4 Gy/28 fractions* 5FU + LV/capecitabine/ capecitabine + irinotecan	Dworak regression grade	95 genes: TYMS and RAD23B	Precision 87%, sensitivity 100%, specificity 82%

Rimkus et al. 2008 [12]	Tumor tissue biopsy	43	No	*45 Gy* 5FU	Becker regression grade	42 genes (i) Overexpressed: CASP1, SLC35E1, CCNK, STAT2, and ETS2 (ii) Downregulated: TDE1, USP42, M-RIP, and FREM1	Accuracy 81%, sensitivity 71%, specificity 86%, PPV 71%, NPV 86%

Nishioka et al. 2011 [13]	Tumor tissue biopsy	17	3	*40 Gy/20 fractions* S1	Japanese Classification of Colorectal Carcinoma	17 genes: MMP7, MMP14, MMP9, MMP1, MMP16, and RRM1	

Casado et al. 2011 [14]	Tumor tissue biopsy formalin-fixed paraffin-embedded biopsies	25 94		*50.4 Gy/28 fractions* 25 oxaliplatin + raltitrexed 94 different treatment	Dworak regression grade	24 genes: genetic profile of 13: (i) 6 overexpressed: ALDH1A1, CDKN1 1, FOS, RELB, STAT3, and TFF3 (ii) 7 downregulated: BAK, MLH1, TYMS, CKB, GPX2, HIG2, and PH-4	Nonresponders: accuracy 86%, sensitivity 87%, specificity 82%

Palma et al. 2013 [8]	Blood sample	27	8	*50.4 Gy/28 fractions* Capecitabine/capecitabine + oxaliplatin	Mandard regression grade	8 genes: FALZ	

Palma et al. 2014 [7]	Tumor tissue biopsy	26	8	*50.4 Gy/28 fractions* Capecitabine/capecitabine + oxaliplatin	Mandard regression grade	257 genes: c-MYC, GNG4, POLA, and RRM1	Accuracy 85%, sensitivity 60%, specificity 100%, PPV 100%, NPV 80%

Gantt et al. 2014 [15]	Tumor tissue biopsy	36	10	*50.4 Gy/30 fractions* 5FU	American Joint Committee on Cancer	183 genes up- and downregulated: RAD50	No response: sensitivity 33%, specificity 100%

Watanabe et al. 2014 [16]	Tumor tissue biopsy	46	16	*50.4 Gy/28 fractions* Tegafur-uracil + leucovorin	Japanese Classification of Colorectal Carcinoma	22 probes (18 genes): signature LRRIQ3, FRMD3, SAMD5, and TMC7	Accuracy 81.3%, sensitivity 100%, specificity 62.5%, PPV 72.7%, NPV 100%

**Table 2:** Studies showing miRNA expression profile predictive of response to CRT in LARC.

Study	Specimen	*N* patients	Validation group	*Radiotherapy dose* Chemotherapy	Response assessment	Identified miRNA: more relevant miRNAs	Outcome
Svoboda et al. 2008 [17]	Tumor tissue biopsies	35		*50.4 Gy/28 fractions* Capecitabine	Dworak regression grade	Interpatient variability miR125b miR137 upregulated during treatment: poor response	

Della Vittoria Scarpati et al. 2012 [18]	Tumor tissue biopsy	35		*45 Gy* Capecitabine + oxaliplatin	Mandard regression grade	57 miRNAs: 13 confirmed by PCR miR-622 and miR-630	Sensitivity 100% Specificity 100%

Kheirelseid et al. 2013 [19]	Formalin-fixed paraffin-embedded biopsies Fresh-frozen biopsies	12		*Not specified* Not specified	Mandard regression grade	Downregulated: miR-10b, miR-143, and miR-145 Upregulated: miR-21 Signature: miR-519c-3p and miR-561	Accuracy 100%

Svoboda et al. 2012 [20]	Tumor tissue biopsy	20		*45 + 5.4 Gy* Capecitabine/5-FU	Mandard regression grade	Nonresponders: Overexpressed: miR-215, miR190b, and miR-29b-2 Lower expression: let7e, miR-196b, miR-450a, miR-450b-5p, and miR-99a	Accuracy 90%

Hotchi et al. 2013 [21]	Tumor tissue biopsy	43	21	*40 Gy/20 fractions* S1	Histopathological RECIST Downstaging	2 miRNAs: miR-223 9 miRNAs: miR-223 3 miRNAs: miR-223	AUC 0.768 Sensitivity 100% Specificity 78%
